# Shared care follow-up of patients with B-cell neoplasms based on nurse-led telephone consultations and PRO-data: a feasibility study from the North Denmark Region

**DOI:** 10.1186/s12913-020-05899-8

**Published:** 2020-11-17

**Authors:** Mia Sommer, Lone Frandsen, Paw Jensen, Søren Ramme Nielsen, Lars Børty Nielsen, Rasmus Froberg Brøndum, Martin Bøgsted, Jakob Madsen, Marianne Tang Severinsen, Erik Elgaard Sørensen, Mette Grønkjær, Tarec Christoffer El-Galaly

**Affiliations:** 1 Department of Hematology, Aalborg University Hospital, Sdr. Skovvej 15, DK-9000 Aalborg, Denmark; 2 Department of Clinical Medicine, Aalborg University, Aalborg, Denmark; 3 Clinical Nursing Research Unit, Aalborg University Hospital, Aalborg, Denmark; 4 Clinical Cancer Research Center, Aalborg University Hospital, Aalborg, Denmark

**Keywords:** Hematologic neoplasms, Feasibility study, Supportive care, Patient-reported outcome measures, Nurse-led telephone consultations

## Abstract

**Background:**

Patients with B-cell neoplasms in remission are monitored with regular physician visits at the hospital. The current standard follow-up procedure is not evidence-based or individualized to patient needs. To improve and individualize the follow-up, we investigated the feasibility of a shared care follow-up initiative, with alternating physician visits and nurse-led telephone consultations and assessments based on patient-reported outcome (PRO) data.

**Methods:**

Patients ≥18 years diagnosed with B-cell neoplasms were eligible for the study when they were in remission and stable without treatment for at least 6 months. Patients were assigned to alternating visits with physicians and nurse-led telephone consultations. The nurse-led telephone consultations were based on PROs, which were collected with the European Organization for Research and Treatment of Cancer questionnaire (EORTC-QLQ-C30), the Myeloproliferative Neoplasm – Symptom Assessment Form, and the Hospital Anxiety and Depression Scale. Patients completed questionnaires before every nurse-led consultation. We also applied the Patient Feedback Form to survey patient acceptance of the requirement of questionnaire completion. We applied descriptive statistics, in terms of counts (n) and proportions (%), to describe the study population and all endpoints.

**Results:**

Between February 2017 and December 2018, 80 patients were enrolled. Adherence, measured as the recruitment rate, was 96% (80/83), and the drop-out rate was 6% (5/80). During the study period, 3/80 (4%) patients relapsed, and 5/80 (6%) patients returned to the standard follow-up, because they required closer medical observation. Relapses were diagnosed based on unscheduled visits requested by patients (*n* = 2) and patient-reported symptoms reviewed by the nurse (*n* = 1). The response rate to questionnaires was 98% (335/341). A total of 58/79 (74%) patients completed the Patient Feedback Form; 51/57 (89%) patients reported improved communication with health care professionals; and 50/57 (88%) patients reported improved recollection of symptoms as a result of completing questionnaires.

**Conclusion:**

Based on patient adherence, a low relapse rate, and positive patient attitudes towards completing questionnaires, we concluded that a shared care follow-up, supported by PROs, was a feasible alternative to the standard follow-up for patients with B-cell disease in remission.

## Background

B-cell neoplasms include diseases, such as non-Hodgkin lymphoma, Hodgkin lymphoma, multiple myeloma, and chronic lymphoblastic leukemia (CLL) [1]. These diseases originate in the lymphatic system, and relevant treatments include chemotherapy, with or without immunotherapy, stem cell transplantation, and novel targeted therapies [1–3]. Patients with incurable B-cell neoplasms that do not require immediate, active treatment are sometimes managed with watch and wait (WAW) follow-ups. These patients, and patients in remission after treatment are routinely followed by the attending physician. Clinical guidelines exist for assessing survivors of hematological cancer during follow-up, but the guidelines are inconsistent [1–3]. The follow-up interval and the total post-treatment follow-up time for patients with B-cell neoplasms depend on the specific diagnosis and whether the patient has curable, aggressive lymphoma or chronic, incurable B-cell malignancies, such as indolent lymphoma or CLL. The follow-up strategy can also be influenced by the choice of therapy and the response to treatment; for example, patients at high risk of relapse are closely monitored, due to the potential for suboptimal response to treatment [4]. In Denmark, patients with CLL or indolent lymphoma that are managed with WAW are followed at the outpatient clinic every 4–12 months, and the frequency depends on a number of clinical parameters [5–7]. For example, when patients with diffuse large B-cell lymphoma (DLBCL) respond to a curative intent treatment with complete remission and have a low risk of relapse, they are followed every 3–4 months in the first year after treatment and every 6 months in the following 2 years; after that, follow-up is terminated. In contrast, patients with a high risk of relapse are typically followed at the outpatient clinic with regular visits for up to 5 years. Similar schedules are applied to patients that receive palliative therapy for incurable B-cell malignancies, but as a rule, follow-ups are lifelong for this group [3]. Post-treatment follow-ups are mainly focused on symptom assessment and physical examinations for signs of recurrent disease [4, 8]. Patient-reported symptoms are the single most important factors for early detection of recurrent lymphoma, as shown in a previous study, where patient-reported symptoms preceded confirmation of lymphoma relapse in 64% of patients [9]. Follow-up strategies for B-cell neoplasms are based on expert consensus and observational studies, due to the absence of supporting evidence from controlled clinical trials [5–7, 10]. Many patients experience frequent, non-specific symptoms, treatment-related complications, and other health-related issues associated with cancer survivorship [11–14]. For example, many cancer survivors experience significant fatigue, neuropathy, and anxiety related to a fear of recurrence [15, 16]; all of these symptoms can have a negative impact on the quality of life [13]. Furthermore, the literature has demonstrated that these patients have reported unmet needs, in particular, emotional and informational needs [17–19]. In recent years, nurse-led interventions have proven successful, in terms of meeting patient needs and addressing psychosocial issues. Additionally, these interventions have demonstrated high patient acceptance and offered advantages, including convenience and individualized care [20–22]. Studies have indicated that PROs can be successfully implemented during the follow-up of patients with cancer; PROs have led to improved patient-provider communication, patient satisfaction, and early relapse detection [23–26]. Moreover, implementing PROs in clinical practice could also support patients in taking an active part in follow-ups and identifying specific health complaints relevant to the individual patient [27, 28]. Hence, combining nurse-led interventions and collecting PROs on cancer survivorship for patients with hematological cancer might contribute to providing patient-centered care and increase the quality of care. This approach has not been sufficiently explored in a hematological setting. Therefore, we investigated the feasibility of a shared care follow-up initiative, with alternating standard physician visits and nurse-led telephone consultations, based on the PROs of patients with B-cell neoplasms.

## Methods

### Study design

This study was designed as a feasibility study. It was conducted from February 2017 to June 2019. Patient recruitment ended in June 2018, and data collection was set to end in December 2018.

### Study location

The study was conducted at the Department of Hematology, Aalborg University Hospital, Denmark. Over the past decade, the number of new patients admitted to this hospital has increased yearly. In 2019, the outpatient clinic served approximately 4000 patients with all varieties of hematological diagnoses, at all stages of the disease trajectory (diagnostics, treatment, and follow-up). Among these patients, approximately 1500 (37.5%) were diagnosed with B-cell neoplasms.

### Study population

Eligible participants included all patients with B-cell neoplasms that were placed under observation in WAW mode or were previously treated and were currently in post-treatment follow-up. The patients were enrolled by their attending physician at a scheduled in-hospital follow-up visit. The sample size was reached by convenience sampling, and as many eligible patients as possible were included during the recruitment period [29]. All patients provided written signed consent before entering the study.

### Study criteria

Inclusion criteria were: age ≥ 18 years; a diagnosis of B-cell neoplasm (CLL and lymphomas); disease in remission or stable without treatment for at least 6 months prior to inclusion; sufficient self-care to report new symptoms; and a willingness to return questionnaires on a regular basis. In this context, and based on the WHO’s definition of the concept, self-care was defined as the ability of an individual patient to seek hospital care when necessary [30]. Exclusion criteria were: medical conditions, such as severe comorbidities that required close medical attention; conditions that impaired the patient’s ability to understand and comprehend the study concept (e.g., dementia); and an inability to complete the questionnaires online.

### Follow-up structure

The nurse-led telephone consultations replaced every other visit with the physician. At the end of every scheduled physician visit, two future consultations were booked, which included one nurse-led telephone consultation and a subsequent in-hospital physician visit. The nurse-led telephone consultations were scheduled ad hoc, and the total number of consultations for each patient was determined by the attending physician, based on the individual follow-up interval and the patient’s diagnosis, risk profile, age, and prior treatment. The physician visits were conducted by the three hematologists that participated in the study. The telephone consulting nurse had 15 years of clinical hematology experience.

The patients completed questionnaires at inclusion and prior to every nurse-led consultation. Patients were also asked to complete the questionnaires prior to physician visits, to monitor health-related quality of life (HRQoL) and symptom burden over time; however, physicians were not obliged to review the responses. Patients accessed the questionnaires from an email-alert with a link to an online healthcare platform (Dansk Telemedicin A/S, Denmark). The email-alert was sent 1 week prior to each scheduled nurse-led telephone consultation; when no response was received to the initial email-alert, a reminder was sent after 3 days. The nurse reviewed responses to the individual questions and the overall scores, which were calculated based on questionnaire-specific standard calculations [31–33]. Based on the questionnaire responses, the nurse assessed any development or abnormal response and discussed solutions and health-promoting options with the patient during the following telephone conversation. When any health issues were reported that might have been related to the B-cell neoplasm, or when other significant health issues emerged, an in-hospital nurse consultation could be arranged the subsequent day. Alternatively, the patient could be offered a physician consultation at the earliest available time slot. Prior to all types of consultations, routine blood tests were assessed by the physicians.

### PRO instruments

PRO data were collected with three instruments: the European Organization for Research and Treatment of Cancer Quality of Life Questionnaire (EORTC-QLQ-C30), the Myeloproliferative Neoplasm – Symptom Assessment Form (MPN-SAF), and the Hospital Anxiety and Depression Scale (HADS) [34–36]. EORTC-QLQ-C30 was a 30-item instrument that included an overall Global Health Status domain, five functional domains (physical, emotional, cognitive, social, and role functioning), and nine symptom domains (fatigue, nausea and vomiting, pain, dyspnea, insomnia, appetite loss, constipation, diarrhea, and financial difficulties). Each domain was scored from 0 to 100, and higher scores indicated better HRQoL, better functioning, or a worse symptom burden [37]. MPN-SAF was a 17-item disease-specific symptom questionnaire developed for patients with myeloproliferative diseases [35]. This instrument provided sufficiently generic questions on symptoms relevant to B-cell malignancies; however, no lymphoma-specific questionnaires were available in the local language at the time the study started. Therefore, we added four ad hoc questions to MPN-SAF to ensure that typical B-cell neoplasm symptoms were covered adequately. The ad hoc questions are presented in Table 1. HADS [36] was a 14-item questionnaire designed to assess anxiety and depression symptoms in patients with somatic diseases. HADS included two scales, one for anxiety (HADS–A) and one for depression (HADS–D), to differentiate the two states. Formal licenses for EORTC-QLQ-C30 and HADS were obtained prior to study start [38, 39]. No formal license was required for MPN-SAF, however, written consent for the use of the questionnaire was obtained from the developer [32].

**Table 1:** Ad hoc questions added to MPN-SAF to cover B-cell neoplasm-specific symptoms associated with recurrent disease in a shared care follow-up initiative

Have you since last consultation:		
**Noticed swollen lymph nodes?**	Yes:	No:
**Had infections that demanded antibiotic treatment?**	Yes:	No:
**Experienced the same symptoms as last time you were ill from your blood disease?**	0 (No) 1 2 3 4 5 6 7 8 9 10 (Yes)	
**Do you feel ill from your blood disease?**	0 (No) 1 2 3 4 5 6 7 8 9 10 (Yes)	

### Patient acceptance

We assessed how well patients accepted the task of completing questionnaires as part of follow-up in a cross-sectional survey, conducted between November 2018 and March 2019. Patients were sent an email invitation to participate via the REDCap survey tool [40] . Data were collected with the Patient Feedback Form (PFF), a generic questionnaire that included 13 items for evaluating the applicability and value of PROs in clinical practice. PFF has been translated into Danish and validated by *Tolstrup et. al.* [41] and written consent to use the questionnaire was obtained from the authors [41]. All participants, including patients that had dropped out of the study for various reasons, were invited to complete the survey. This survey was conducted anonymously to limit a potential response bias and to avoid an overestimation of patient acceptance [42].

### Statistical analyses

The endpoints were patient inclusion, the questionnaire response rate, the drop-out rate, and patient acceptance of completing questionnaires as a part of the follow-up. We performed descriptive statistics, expressed as the counts (n) and proportions (%).

## Results

### Patient adherence

**Fig. 1:**
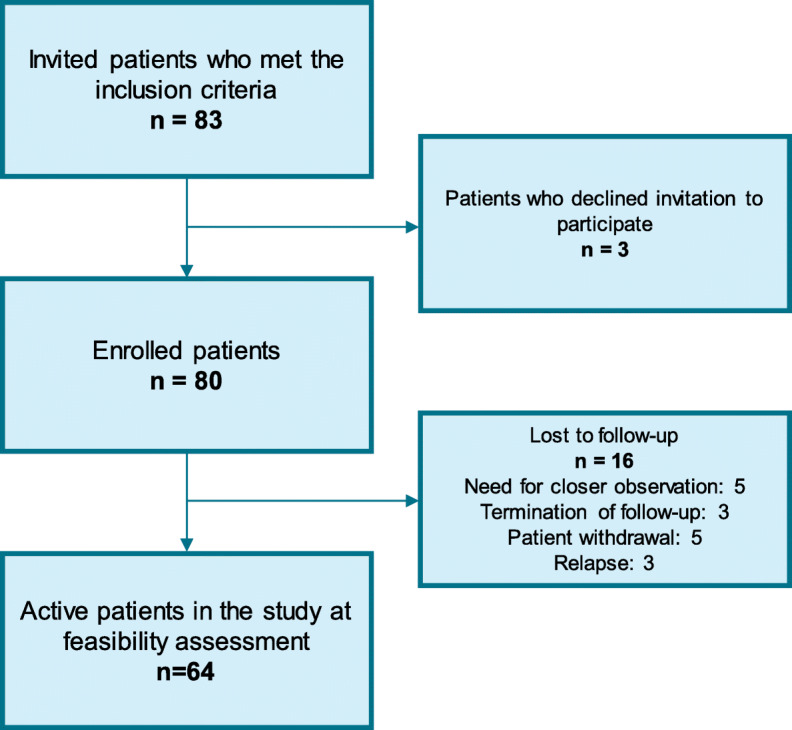
Flowchart of the patient inclusion process

Eighty-three patients were invited to participate in the study. The patient inclusion process is illustrated in Fig. 1. Among these, 80 patients agreed to participate; thus, the recruitment rate was 96% (80/83). Among the 80 included patients (median age 68 years, range 28–82); 42/80 (53%) were men and 38/80 (48%) were women. The most frequent B-cell neoplasms were follicular lymphoma (FL; *n* = 29), CLL (*n* = 17), and DLBCL (*n* = 14). Relevant characteristics of the study population are shown in Table 2. During the study period, 341 questionnaires were distributed. Of these, 335 were completed and returned, which resulted in a response rate of 98% (335/341).

**Table 2:** Baseline demographics, treatment, and follow-up characteristics of patients with B-cell neoplasms included in a shared care follow-up initiative

Characteristic	Total
**N**	80
**Female**	38
**Male**	42
**Age, y; median**	68
**Age range, y**	28–82
**Diagnosis**	
*FL*	29
*DLBCL*	14
*CLL*	17
*MZL*	10
*WM*	5
*LPL*	3
*MCL*	1
*HL*	1
**Number of visits to a physician, median (range)**	1 (0–4)
**Number of nurse consultations, median (range)**	1 (0–4)
**Time since diagnosis, y; median (range)**	4.4 (1.1–20.2)
**Time since last treatment, y; median (range)**	3.4 (1.3–16.3)
**Follow-up interval, months**	
*2–3*	34
*4–5*	25
*6*	21
**Number of treatment lines**	
*1*	48
*2*	4
*> 2*	4
*No previous treatment (watch and wait)*	24

*FL* Follicular Lymphoma, *DLBCL* Diffuse Large B-Cell Lymphoma, *CLL* Chronic Lymphocytic Leukemia, *MZL* Marginal Zone Lymphoma, *WM* Waldenstrom Macroglobulinemia, *LPL* Lymphoblastic Lymphoma, Mantle Cell Lymphoma, *HL* Hodgkin Lymphoma

During follow-up, 5/80 (6%) patients dropped out of the study due to excessive questions asked (*n* = 1), impaired eyesight, which led to difficulties reading the questionnaires (*n* = 1), and a preference for regular hospital follow-ups (*n* = 3). Another 8/80 (10%) patients dropped out of the study due to medical reasons. Of these, 3/80 (4%) patients experienced a relapse of the B-cell neoplasm. Two relapses (DLBCL) were diagnosed at unscheduled visits requested by the patients, due to the symptoms experienced. The other relapse (MZL) was detected when patient-reported symptoms were reviewed by the study nurse prior to the nurse consultation. Finally, 5/80 (6%) patients were excluded because closer medical observation was needed due to mental problems (*n* = 1), infections unrelated to B-cell neoplasia activity (*n* = 2), suspicion of relapsed disease raised by an enlarged lymph node, which required regular manual assessments (*n* = 1), and terminal illness, due to another cancer (*n* = 1).

### Nurse-led telephone consultations

During the study period, 129 nurse-led telephone consultations were conducted (median 1 consultations/patient, range 1–4) and the median time spent in a nurse consultation was 12 min (range 3–67 min). Forty-six patients 46/80 (58%) were followed with one or more nurse-led telephone consultations without further action, and 34/80 (43%) patients had blood test results or a symptom burden that required discussion with the physician. Five patients 5/34 (15%) were scheduled for an in-hospital appointment with their attending physician after a discussion between the nurse and the attending physician. Twenty-two patients 22/80 (28%) were advised to seek help from their general practitioner, due to health complaints that were determined to be unrelated to B-cell neoplasia (e.g., dyspnea, urinary issues, musculoskeletal pain). No patients were scheduled for a nurse visit at the outpatient clinic. In total, 124/129 (96%) nurse-led telephone consultations replaced a visit to the hospital, compared to the standard follow-up procedure.

### Patient acceptance

We conducted a patient acceptance survey to assess attitudes towards completing questionnaires as part of the follow-up. One patient died prior to the survey start, hence 79/80 (99%) patients were invited to participate. Among these, 58/79 (74%) patients agreed to participate, including 24/56 (43%) women and 32/56 (57%) men (2 patients did not provide gender information). The patient feedback suggested that patients had an overall positive attitude towards completing questionnaires as part of the follow-up. The results from the patient acceptance survey are presented in Table 3.

**Table 3:** Patient Feedback Form results on patient acceptance of completing questionnaires as part of a shared care follow-up initiative for patients treated for B-cell neoplasms

Questions	Number of respondents	Responses, N (%)			
		Too short	Just right	Too long	
**The lengths of the questionnaires were**	58	0 (0)	47 (81)	11 (19)	
		**Not often enough**	**Just right**	**Too often**	
**The number of times I was asked to complete the questionnaires was**	58	0 (0)	53 (91)	5 (9)	
		**Strongly agree**	**Agree**	**Disagree**	**Strongly Disagree**
**It was easy to complete the questionnaires**	58	15 (26)	36 (62)	7 (12)	0 (0)
**It made sense to complete the questionnaires**	58	16 (28)	36 (62)	5 (9)	1 (2)
**It was easy to understand the questions**	58	18 (31)	36 (62)	4 (7)	0 (0)
**Completing the questionnaires made it easier for me to remember my symptoms and side effects, when I spoke with the healthcare professionals**	57	15 (26)	35 (61)	7 (12)	0 (0)
**Completing the questionnaires improved the conversation with the health care professionals**	57	14 (25)	37 (65)	6 (11)	0 (0)
**The health care professionals used the information from the questionnaires in connection with my treatment**	54	15 (28)	30 (56)	9 (17)	0 (0)
**It is my experience that the quality of my treatment was improved due to the fact that I had completed the questionnaires**	54	9 (17)	35 (65)	10 (19)	0 (0)
**It is my experience that the communication with the health care professionals was improved due to the fact that I had completed the questionnaires**	55	9 (16)	35 (64)	11 (20)	0 (0)
**Completing the questionnaires made me feel involved in my treatment**	56	18 (32)	30 (54)	8 (14)	0 (0)
**I would recommend that other patients should complete the questionnaires**	56	23 (41)	32 (57)	1 (2)	0 (0)
**I would like to continue completing questionnaires in the future**	54	24 (44)	24 (44)	4 (7)	2 (4)

## Discussion

This study investigated the feasibility of a shared care follow-up initiative, featuring alternating nurse-led telephone consultations, supported by PROs, and regular physician visits, as an alternative to the standard follow-up for patients treated for B-cell neoplasms. In this study, we found a high recruitment rate of 96% (80/83) and a high questionnaire response rate of 98% (335/341). *Saltbæk* et al., tested the feasibility of a nurse-led follow-up among survivors of breast cancer. They reported a recruitment rate of 78% and a questionnaire response rate of 95.3% [43]. Furthermore, *Beaver* et al.*,* tested the feasibility of nurse-led telephone consultations in survivors of prostate cancer. They reported a recruitment rate of 75% [44]. Hence, our findings were consistent with previous feasibility studies of nurse-led telephone consultations. In fact, our recruitment and questionnaire response rates were higher than those reported in earlier studies. These results suggest that nurse-led telephone consultations supported by PROs are feasible among survivors of B-cell cancer. During the study period, five patients withdrew from our study, due to various reasons, which led to a drop-out rate of 6% (5/80). Similarly, *Saltbæk* et al.,and *Beaver et a.l,* reported drop-out rates of 4 and 6.5%, respectively [43, 44], consistent with our findings. These results suggest that survivors of hematological cancer could adhere well to a follow-up model that included nurse-led telephone consultations and PROs. The patients that withdrew from the study varied in age, gender, and diagnosis, which suggested that dropping-out was not driven by specific characteristics. Additionally, only 3/80 (4%) patients relapsed during the study period, which was a low relapse rate in the selected population. Interestingly, two relapses were discovered by patients. One had clear symptoms that were reported in the questionnaires underscoring the ineffectiveness of routine follow-up in detection of relapse in patients with low risk of relapse. This is consistent with the findings in a recent Danish study showing that patient reported symptoms prereceded relapse detection in the majority of the patients with DLBCL [45]. In total, 10/80 (10%) patients left the study during the study period due to relapse, a need for closer observation or as a consequence of the patients’ choice. Thus, despite targeting patients with high degree of self-care and low risk of relapse, follow-up should be a dynamic process and allow patients to shift between follow-up strategies according to preferences at a given time.

The patients’ feedback suggest that most patients found it easier to recall relevant symptoms and found that the conversations with health professionals improved having returned questionnaires. This finding was consistent with those of *Greenhalgh* et al., who showed that PROs in clinical practice could help support patient-provider communications and patient care. The authors concluded that completing PROs could potentially change the way patients reflected on their health condition [26]. Indeed, we found that, overall, patient acceptance was positive. Although our findings on patient acceptance might not directly reflect the feasibility of the initiative, the patient acceptance did reflect patient perspectives on completing questionnaires as a part of clinical practice.

Our findings on the feasibility of nurse-led telephone consultations were consistent with similar initiatives, which also showed that nurse-led telephone consultations were feasible, efficient, and well-accepted by patients [20–22, 46]. Most previous studies focused on patients with solid tumors, such as breast, prostate, and/or lung cancer. However, one previous study investigated the feasibility of nurse-led telephone follow-up for patients with indolent and chronic hematological malignancies [47]. Another study tested a model for following-up patients that survived lymphoma [48]. The present study has added evidence to existing studies with our findings that a high recruitment rate and high response rate gave rise to the positive patient adherence in patients that survived hematological cancer. Furthermore, we presented a follow-up model that was based on nurse-led telephone consultations, but with the added requirement that PROs were actively used as a tool for the detection of present health concerns. The systematic use of PROs as preparation for the nurse-led telephone consultations made it possible to provide problem-based consultations and address specific patient concerns at an appropriate time. In addition, the PROs served to prepare the patients for their upcoming consultations, in terms of their awareness of new or persistent symptoms. Consequently, the active, systematic use of PROs prior to any patient contact appeared to be valuable in clinical practice. However, the potential value of the use of PROs should be considered in light of the fact that this joint initiative included both the option to rearrange the follow-up and the addition of PROs; hence, we should not draw conclusions on these components separately. However, the patient feedback suggested that PROs could be valuable, both in clinical practice and in a shared care follow-up initiative, because they enhanced patient reflection on their present symptoms, patient-provider communications, and patient involvement.

Although we found that this follow-up model was feasible, and we observed positive patient adherence, the study was conducted without a control group. Therefore, we could not conclude whether this initiative improved the quality of care or patient satisfaction over the standard follow-up.

It has been demonstrated that some patients declined participation in telehealth interventions, because they felt unsure about using technology or they had concerns about privacy [49, 50]. This suggested that a large number of declined invitations to participate could contribute to an overestimation of the feasibility of the intervention. In the present study, we did not determine the reasons for declining participation. Although this is important knowledge, only three patients declined participation after receiving detailed information about the study by the study nurse. This low decline rate suggested that the concerns of those patients did not significantly influence our estimation of study feasibility.

In this study, the patients were selected by their attending physicians, which might have contributed to that the patients’ refrained from declining participation. However, in order to limit potential influence from the physician – patient relationship, strong emphasis was placed on the fact that participation was voluntary and that the patients could return to in-hospital follow-up without any consequences for future treatment and care.

The sample size was achieved by convenience sampling [29, 51]; we included as many patients possible during the study period. The shared care follow-up initiative was offered as an optional service for interested patients, and it did not plan to include patients that felt more secure with in-hospital visits. However, convenience sampling poses the risk of a selection bias. The main assumption of this sampling method is that the target population is homogeneous, compared to random sampling methods [29, 51]. Consequently, our sample might have been too homogeneous to generalize results to other patient populations. However, by including 80 patients, we attempted to increase the likelihood of including a heterogeneous study population [52]. Indeed, some heterogeneity was achieved, based on the age range of the included patients and the range of disease types. However, the size of the study population was insufficient to provide powered, meaningful subgroup analyses of individual diagnostic categories or age groups. Furthermore, the limited drop-out rate did not allow an exploration of the features associated with the likelihood of completing the study.

## Conclusion

To provide evidence for revising the standard follow-up procedure, we tested the feasibility of a shared care follow-up initiative, supported by PROs, for patients with B-cell neoplasms. We found good patient adherence, in terms of a high recruitment rate, a high response rate, and a low drop-out rate. Furthermore, relapse detection did not seem to be compromised. Patient feedback suggested that patients had an overall positive attitude towards completing questionnaires as part of the follow-up. In conclusion, a shared care follow-up initiative supported by PROs for patients with B-cell neoplasms in remission appeared to be feasible and acceptable as an alternative to standard practice for a patient population with a perceived high level of self-care and low risk of relapse. The inclusion of PROs in the post-treatment follow-up was a valuable addition, in terms of increasing reflection on the patient’s own health, enhancing patient-provider communications, and encouraging patient involvement. Although further research is warranted, including an exploration of patient experiences, based on our results, the systematic use of PROs could potentially offer a more individualized, problem-based approach to nurse-led consultations and provide support for more meaningful, relevant physician consultations. Future studies should explore the cost effectiveness of this proposed model of shared care follow-up. In addition, other aspects of PRO use should be evaluated in the follow-up of patients with B-cell neoplasms, such as the potential for promoting early detection of relapse or progressive disease in a more high-risk population.

## Data Availability

The datasets used and/or analyzed during the current study are available from the corresponding author on reasonable request.

## Abbreviations

PRO: Patient-reported outcome
EORTC-QLQ-C30: The European Organization for Research and Treatment of Cancer – Quality of Life Questionnaire – 30 questions
MPN-SAF: Myeloproliferative Neoplasm – Symptom Assessment Form
HADS: Hospital Anxiety and Depression Scale
PFF: Patient Feedback Form
CLL: Chronic Lymphocytic Leukemia
WAW: Watch and wait
HRQoL: Health-related quality of life
FL: Follicular Lymphoma
DLBCL: Diffuse Large B-Cell Lymphoma
